# The Book World of Medicine and Science

**Published:** 1901-04-20

**Authors:** 


### The Book World of Medicine and Science.

The Present Position of the Treatment of Simple Fractures of the Limbs. By William H. Bennett, F.R.C.S. (London: Longmans, Green and Co. 1900. Pp. 41. Price 2s. 6d.)

This essay from the pen of Mr. Bennett, when delivered in its original form at the Ipswich meeting of the British Medical Association, deservedly evoked much attention and provoked much discussion. It is now before us in a revised form, and is accompanied by an appendix summarising the opinions and practice of about 300 surgeons of experience. The subject is of such importance as to deserve a more critical analysis than is possible in the brief space of a review, and we can at present do little more than attend to the important points raised by Mr. Bennett. He finds, in the first place, that the treatment of simple fracture is in a state of transition at the present time, and that the tendency of surgeons is to employ what are called "rational methods" instead of prolonged splinting. It is equally certain that in not a few institutions certain methods of treatment have been traditionally employed for many years with but little change, and that practitioners educated at these institutions adopt persistently these traditional methods. A few—a very few—surgeons consider most fractures of long bones suitable for treatment by operation. Many surgeons on the other hand see no necessity or justification for operation under such circumstances. But most practitioners, whilst willing under certain conditions and particular circumstances of the patient and his injury to adopt operative procedures, under ordinary circumstances adopt non-operative methods. These methods fall into three groups: (*a*) The application of immovable splints or apparatus; (*b*) the application of splints removable for examination or passive movement; (*c*) the rejection of splints for any considerable period. The increase of late in the number of surgeons who have early resort to passive movement or to massage has been followed by a natural tendency to discard plaster splints and to resume the use of movable splints. In spite of the persuasive eloquence of several surgical enthusiasts, the general opinion of practitioners is, Mr. Bennett finds, adverse to operation in all save two varieties of simple fracture, namely, fracture of the olecranon and of the patella. It would further appear that however excellent a method under particular circumstances that of immediate suture of the patella may be, yet it does not commend itself as routine treatment to general practitioners. And the general opinion seems to be that, in all but a few cases, after fracture of an olecranon a perfect result may be obtained without operation. Of massage and early movement in recent fractures fully 50 per cent. of London and 70 per cent. of country practitioners have had no experience. But of those who have had experience the majority speak in terms of approval. A minority are, it must be confessed, entirely opposed to it; one surgeon, for example, has seen two cases of embolism arising in the course of treatment and therefore considers it dangerous.

An important point on which there seems to be general agreement is that disability following fracture is more often due to matting of parts about the fracture and joints than to faulty union of the bones, and this is deserving of special attention in connection with the question of massage and passive movement.

We have been only able to indicate a few of the important matters alluded to by Mr. Bennett. His essay and the statistical appendix will repay careful perusal, and we consider that the carrying out of this tedious investigation into the opinions of 300 surgeons, and the critical examination of the tabulated results, is not the least of Mr. Bennett's many services to surgery.

Surgical Regulations, Great Yarmouth Hospital.

We may congratulate the anonymous author of this useful little guide to operating-room procedure. Naturally the arrangements must vary at different hospitals, but it is clear that wherever nurses, drawn perhaps from different training schools, have to work together, it is essential that some definite rules on the subject should be laid down to prevent confusion, and this purpose seems to be well served by the pamphlet before us. It is apparently drawn up for a hospital where a steam steriliser is not in use, which perhaps accounts for some of the provisions laid down; but as a guide for smaller hospitals this is all the better, as showing what can be accomplished by mere boiling and the use of antiseptics. It seems to be the intention of the author that the sponges and swabs should be taken by the surgeon direct out of the lotion in which they are handed to him, a proceeding which involves the frequent application of the lotion itself to the wound—a thing which many surgeons object to. This may possibly be difficult to avoid when sponges are used, but when gauze mops are employed it seems to be a better plan that they should stand in a pile, as dry as may be, either fresh from the steam steriliser or freshly wrung out of the water in which they have been boiled. There can be no doubt that nurses trained to work together on a prearranged plan such as is laid down in this book, each knowing her own place and her own duties, will make far more useful and efficient helpers than even the most intelligent nurses working each on her own initiative, or trusting to the directions given on the spur of the moment. We do not say that every hospital ought to adopt precisely these regulations, but certainly every hospital ought to have something of the same sort; a definite, preordered plan for the conduct of the accepted surgical technique.

The Medical Annual: a Year-Book of Treatment and Practitioners' Index. 1901. Nineteenth year. (Bristol: John Wright and Co. Price 7s. 6d. net.)

Again we have to welcome the appearance of this most useful annual, which, while following on the old lines on which the wide popularity of the book has been built up has kept well to its tradition of giving a good account of everything new which has appeared during the year. It is this happy combination of the handbook of treatment with the record of progress which makes the "Medical Annual" so useful a practitioner's companion—a book quite different from any other work we know of. Among the special features of this issue are articles on "Toxins and Antitoxins," "The Light Treatment," "X-Ray Work in Medicine and Surgery," "Colour Blindness," and other subjects. It is a most useful book, it abounds in illustrations, and is so full of recent information as to occupy a very important place on the practitioner's desk.

